# Vehicle emission and atmospheric pollution in China: problems, progress, and prospects

**DOI:** 10.7717/peerj.6932

**Published:** 2019-05-16

**Authors:** Jin Wang, Qiuxia Wu, Juan Liu, Hong Yang, Meiling Yin, Shili Chen, Peiyu Guo, Jiamin Ren, Xuwen Luo, Wensheng Linghu, Qiong Huang

**Affiliations:** 1 School of Environmental Science and Engineering, Guangzhou University, Guangzhou, Guangdong, China; 2 Department of Geography and Environmental Science, University of Reading, Reading, United Kingdom; 3 Collaborative Innovation Center of Atmospheric Environment and Equipment Technology, Jiangsu Key Laboratory of Atmospheric Environment Monitoring and Pollution Control, School of Environmental Science and Engineering, Nanjing University of Information Science and Technology, Nanjing, Jiangsu, China; 4 College of Chemistry and Chemical Engineering, Shaoxing University, Shaoxing, Zhejiang, China

**Keywords:** Vehicle emission, Air pollution, PM2.5, Oil, Road, Renewable energy, Regulatory innovation

## Abstract

China has been the largest vehicle market in the world since 2009. The stalemate between the rapid development of the vehicle industry and delayed vehicle emission control has become increasingly prominent. Vehicle emission has become a significant source of air pollution in China’s cities. Understanding the current barriers in the vehicle industry is necessary for the development of effective and sustainable measures and policy to manage vehicle-induced air pollution. This review provides insight into the circumstances and causes of vehicle-induced air pollution and outlines recent progress in policy-makers’ long-term strategies and regulations. The development of an integrated mechanism of social participation, technical revolution, and regulatory innovation in vehicles, fuel, and roads is suggested to break the stalemate between air pollution and the automobile boom in China; the implications of this review extend to other countries facing the similar atmospheric pollution problems.

## Introduction

Atmospheric pollution has emerged as one of the primary environmental issues in China and other developing countries. Despite the slow development of the automobile industry before the 1990s, China experienced rapid growth in new vehicle sales, especially after becoming a member of the World Trade Organization (WTO) in 2001. By 2009, China had the largest vehicle market in the world (Wu et al., 2016). Consequently, the vehicle industry has become an increasingly significant contributor to Gross Domestic Product (GDP) in China. On the other hand, China ranked the 128^th^ out of 133 countries with a score of 22.3 in overall air quality (Zhao, 2014). Moreover, according to the 2016 Environmental Performance Index released by Yale and Columbia University at the World Economic Forum, China ranked the 109^th^ out of 180 countries (Liu et al., 2017b). The poor environmental quality is due to a variety of factors, most notably the serious air pollution, which presents a significant technological, legal, and regulatory challenge (Yang, 2014). Current environmental policies and measures on air pollution are far behind the development of the vehicle industry in recent decades. Air quality in China, especially in many cities, has received unprecedented attention since the serious PM_2.5_ (particulate matter no larger than 2.5 µm in diameter) pollution incidents in Beijing in 2013 (Song et al., 2018; Zhu, 2018). Along with other pollutants, researchers and the public have increasingly considered vehicle emissions to be a significant factor in deteriorating air quality.

On both regional and national scales, vehicle emission has been identified as one of the most important contributors to air pollution in most cities in China (Gong et al., 2017; Wang et al., 2010; Wu et al., 2016), and has even surpassed other contamination sources in some cities. Vehicle emission from the consumption of a large amount of fossil fuel has been identified as a main contributor to air pollution in China (Huang et al., 2016a), and also a major source of greenhouse gas emission (Feng, Chen & Zhang, 2013). Beijing-Tianjin-Hebei (BTH), the Yangtze River Delta (YRD), and Pearl River Delta (PRD) have emerged as China’s most advanced industrial zones and the most densely populated regions in China (Li et al., 2018; Meng et al., 2018). They are facing unprecedented pressure to mitigate vehicle emissions and improve regional air quality (Guo et al., 2016; Li et al., 2018; Liu et al., 2017c; Yang et al., 2016; Zhang et al., 2017). Air pollutants are associated—either directly or indirectly—with motor vehicle emissions in the form of carbon monoxide (CO), nitrogen oxides (NOx), hydrocarbons (HC), volatile organic compounds (VOCs) and particulate matter (PM) (Bell, Honour & Power, 2011; Yang et al., 2015), most of which are primary air pollutants. It is noted that VOCs are often referred to as unburned HC in engines, resulting from incomplete combustion including flame quenching, misfire, detachment of lubricating oil film, and other processes (Samaras & Sorensen, 1998). Studies have shown that vehicle emissions contribute approximately 12–36% of the total NO_X_, 37–43% of the total VOCs, 10.7% of the total PM_10_, and 16.8% of the total PM_2.5_ in China (Che et al., 2011; Liu et al., 2017a; Yin et al., 2015; Zheng et al., 2009). The mixed chemical emissions are responsible for the formation of secondary pollution, such as photochemical smog, visibility reduction, and haze (Wang & Xie, 2009), causing tremendous damage to human health and ecosystems (Yang et al., 2018). NOx can react with HC, VOCs, and other pollutants with ultraviolet radiation to form ground-level ozone and photochemical smog. Therefore, sources and concentrations of NOx and VOCs are of great concern. For example, in the Beijing-Tianjin-Hebei region, the total anthropogenic VOCs emissions accounted for ∼10% while biogenic VOCs emissions contributed ∼28% in 2006 (Li & Xie, 2014). The combined effect of urban anthropogenic emission and rural biogenic sources on regional ozone formation should receive more attention (Yang, Flower & Thompson, 2018; Zong et al., 2018).

An increasing number of studies have indicated that air pollution is an important contributor to a variety of diseases. Epidemiological cohort studies assessing exposure to air pollution are crucial to evaluate the links between air pollutant exposure and health effects (Vedal et al., 2017). Traffic-related emissions contain different kinds of air pollutants such as ultrafine particles, nitrogen oxides, and diesel soot that are related to mortality risks (Van Wijnen & Van der Zee, 1998). The cohort study confirmed that exposure to diesel fueled vehicle emissions increased mortality risk from lung cancer (Attfield et al., 2012). In China, studies found a clear relation between air pollution and the increasing number of cases of respiratory diseases. Bai et al. (2018) found a significant relationship between traffic-related air pollution and hospital visits for childhood Acute Bronchitis (AB), particularly in school-age children during cold seasons. It was found that an interquartile range increase in concentrations of NO_2_, PM_2.5_, and CO would significantly increase the daily hospital visits of childhood AB with 4-day cumulative effect (Bai et al., 2018). However, more cohort studies concerning vehicle emission exposure are required in China.

In spite of its status as a developing country, China has entered the developed auto world. The complex traffic congestion and prominent vehicle emissions could not be solved promptly. To mitigate vehicle emissions, developing a reliable emission inventory of each pollutant is the first step. It is important to accurately measure vehicle emissions from different fuels under various operating conditions (Huang et al., 2016b). An inexact multistage stochastic air-quality model has been developed for controlling multiple pollutants arising from point and mobile sources (Lv et al., 2012). Some researchers advocate the implementation of traffic mitigation measures from economic, technical, and administrative aspects to minimize vehicle emissions (Zhang et al., 2018). In non-ideal conditions, vehicles operate at a low travel speed and often accelerate, decelerate, and idle, yielding more pollutant emissions. Therefore, Hao et al. (2000) suggested that improving road systems can mitigate air pollution from vehicles. Yue et al. (2015) highlighted the importance of upgrading fuel quality to reduce pollutant emissions. Obviously, atmospheric pollution from vehicle emissions in China cannot be easily managed with a single approach. Wearing facial masks can reduce 30%–90% of inhaled air pollutants, and the filtration efficiency depends on the types and quality of the facial masks (Cherrie et al., 2018). The complexity of highly variable traffic emission sources, regional differences in fuel quality, and poor design of road systems all call for an integrated strategic control of vehicle-induced air pollution based on social participation, technical revolution, and regulatory innovation.

In this review, we synthesize an understanding of the situation and analysis of the causes of vehicle emission-associated air pollution in China. The objectives of this study are to (i) analyze the primary causes for the air pollution induced by vehicles emission, (ii) identify the historical barriers and on-going solutions to the stalemate between air pollution and the automobile boom in China, and (iii) propose constructive recommendations for policy-makers to address the challenges of “vehicles, oil, and roads” in the aspects of social participation, technical revolution, and regulatory innovation.

## Survey Methodology

Papers that were reviewed in this research were obtained in journal databases and subject-specific governmental and professional websites. A literature survey was conducted in Web of Science, Google Scholar, Wanfang (Chinese literature database), and CNKI (Chinese literature database) using the following keywords: traffic, car, vehicle, road, highway, motorway, oil, diesel, petrol, or gasoline/diesel combined with air quality, air pollution, particulate matter (PM), ozone, NOx, CO, HC, VOCs, SO_2_, secondary pollution, or emission control. In addition, the websites of the Ministry of Transport, Ministry of Ecology and Environment (the previous Ministry or Environmental Protection) of the People’s Republic of China, and National Petroleum Products and Lubricants Standardization Committee were searched and referenced manually with special attention to obtain any relevant information on vehicle emissions, oil product standards, and vehicle-related air pollution.

## Problems

### Explosive increase of vehicles and caused air pollution

**Figure 1:**
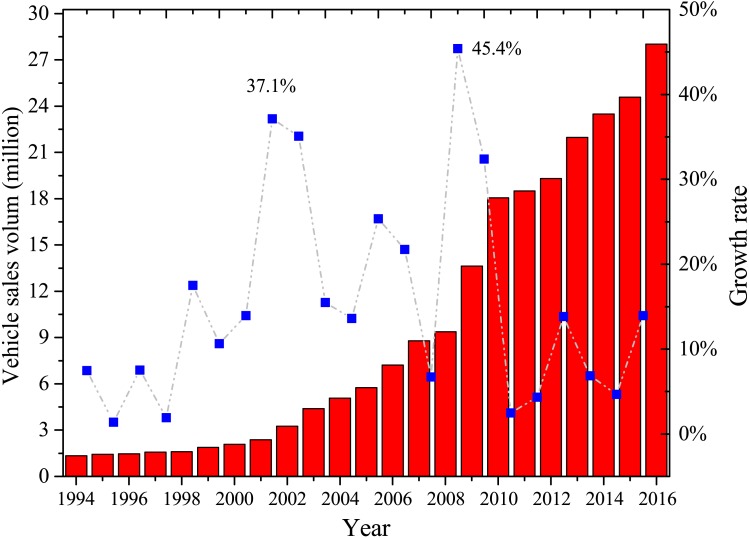
Vehicle sales volume and growth rate in China from 1994 to 2016.

With the rapid development of Chinese economy and significant increases in traffic networks, the number of vehicles has grown at an alarming rate. Annual sales of on-road vehicles have grown from roughly 13.64 million in 2009 to 28 million in 2016 (Chinese Automotive Technology & Research Centre, 2017; Sun, 2017). The growth rate in 2002 and 2009 reached 37.1% and 45.4%, respectively (Fig. 1) (Chinese Automotive Technology & Research Centre, 2015). The former increase occurred due to China’s inclusion in the World Trade Organization (WTO), and purchasing a car is no longer unrealistic due to increased income, particularly in the city middle class. In the 2008 world financial crisis, China’s economy was less affected than many other countries. Moreover, China’s State Council took effective measures to stimulate domestic demand and consumption, and the automobile industry was augmented rapidly. In 2014, China’s automobile productions and sales reached over 23 million and ranked the first place in the world afterward.

In the next decade, the number of private cars will continue to increase in China, especially in some flourishing cities. It seems that the development of the Chinese automobile market is occurring at an alarming rate; however, in reality, per capita vehicle ownership is still lower than those in the US and European countries. There are still many people in China who cannot afford a car. China is still in the process of rapid urbanization at the rate of approximately 1% per year, indicating that about 14 million Chinese citizens move from rural to urban areas each year (Yang et al., 2016; Yang et al., 2014; Zhang & Gong, 2004). Therefore, the development of the Chinese automobile market is still stably developing.

**Figure 2:**
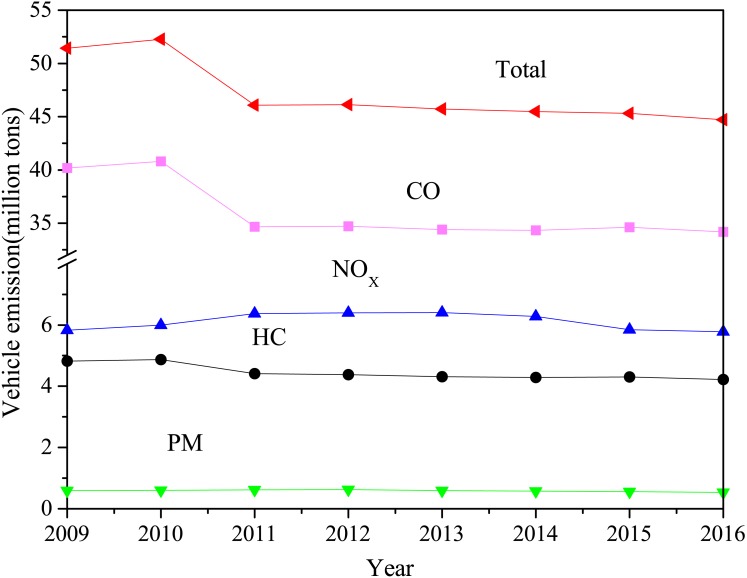
The amount of air pollutants from vehicle in China from 2009 to 2016 (data from China MEP, 2010–2016).

**Figure 3:**
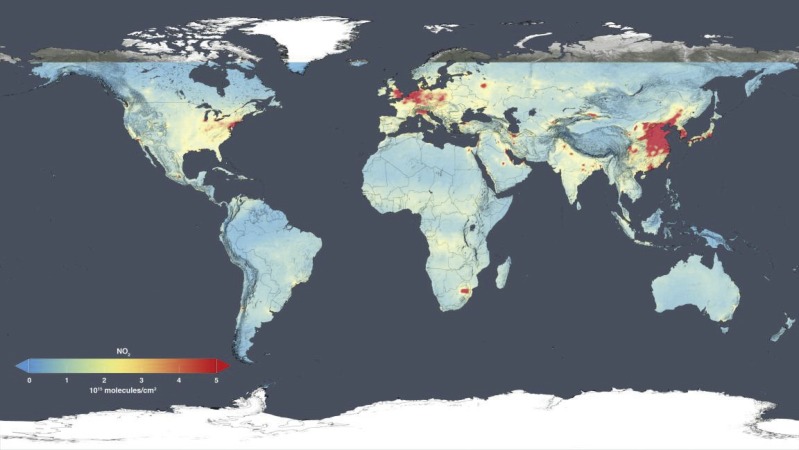
Global map shows the 2014 annual average concentration of nitrogen dioxide in thetroposphere over China overtakes other countries (Source: the National Aeronautics and 725 Space Administration (NASA): https://www.nasa.gov/press-release/new-nasa-satellite-maps-show-human-fingerprint-on-global-air-quality).

The significant number of vehicles and relatively rapid growth rate are major factors contributing to worsening atmospheric pollution. For instance, the huge number of on-road vehicles consumed 95 million tons (Mt) of gasoline and 172 Mt of diesel in 2013 (Wu et al., 2017). It is predicted that the number will continually rise with the increase in car sales. The combustion of gasoline or diesel fuel in vehicle engines produces a variety of harmful chemical emissions. Notably, the emission of CO, HC, NOx, and PM has remained at a high level for many years in China (Ministry of Environmental Protection of the People’s Republic of China, 2017) (Fig. 2). For example, the annual average concentration of NO_2_ in the troposphere over China was much higher than that in the USA and most European countries in 2014 (Fig. 3). Air pollution caused by vehicle emissions also exhibited inter-city variations inside China. The majority (95.9%) of 74 surveyed cities in China failed to meet the PM_2.5_ emission standard, and only 3 cities are notably clearer due to relatively lesser traffic flow and industrial pollution (Song, 2014). Large cities like Beijing and Shanghai attracted extra attention due to their highly dense traffic conditions and comparatively prominent air pollution. For example, the air quality index (AQI) in Beijing soared up to 993 in January 2013, far beyond levels that health experts consider extremely dangerous (Wong, 2013). Some provincial capital cities like Kunming and Lhasa in southwest China performed well; their annual AQI was less than 64 during one-third of 2014 (Xu et al., 2017). Hence, controlling the fast-growing number of vehicles is deemed to be the first crucial step in vehicle emission control.

### Road problem

#### Unreasonable design of road systems

The development of the auto industry brings out the expansion of the road network. However, this also means that road network capacity cannot meet the requirement of the vehicle industry boom. China’s urban road network is usually constructed according to a second-class highway standard, with the advantage of prompt connection to residential areas, commercial areas, and main roads as well as the disadvantage of easily causing severe traffic congestion (Wu, Shan & Wang, 2014). Furthermore, it is unfavorable for short distance travel, but the proportion of short distance travel is quite high in urban areas.

Non-motorized traffic still accounts for a significant proportion of China’s transportation system. For instance, bicycle travel accounts for approximately 36% of the total urban travel in the major cities (Xu, 2000). However, the existing urban road network design in China is dominated by motor vehicles, ignoring the need for non-motorized traffic. Bicycle lanes and sidewalks are attached to the sides of the motorway, and it is common that cyclists and motorists share the non-barrier section in urban roads in China. Furthermore, many Chinese cities still lack a “slow traffic” network including reasonable pedestrian and bicycle lanes, which promotes vehicle use. Other problems include poor “slow traffic” facilities, a shortage of bicycle parking areas, and a lack of a certain number of crossing facilities, such as overpasses, underpasses, and pedestrian crossings. It is essential for stakeholders to set up a rational plan for a non-motorized traffic system. Increase in non-motorized traffic can significantly reduce the traffic volume of motor vehicles and related air pollution.

#### Road construction far behind vehicle increase

**Figure 4:**
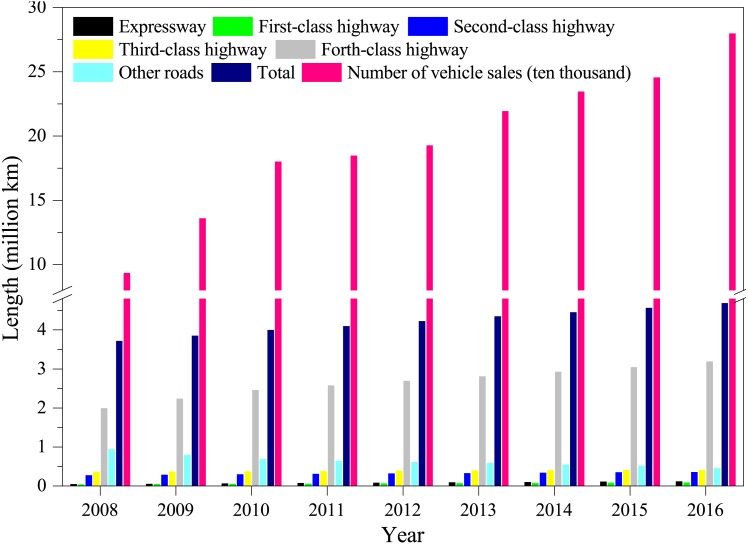
The comparison of total length of different types of roads and vehicle sales in China in the period of 2008–2016.

Currently, transportation infrastructure development has not been able to keep up with the significant increase in the number of vehicles in China (Fig. 4). Taking Guangzhou, the largest mega-city in southern China, as an example, the average vehicle growth rate was more than 20% while the growth rate of road length and capacity was only ∼10% (He, Huo & Zhang, 2002). Slow increases in transportation infrastructure—particularly highway—hindered the mitigation of air pollution caused by vehicle emissions. It is worth noting that the total coverage and increase in highway networks are substantially smaller than other types of roads (Fig. 4), worsening the emission from slow traffic. For example, the average velocity of vehicles on main roads during daytime is below 20 kilometers per hour in Beijing (He, Huo & Zhang, 2002).

### Obstacles to improving fuel quality standards

Impediments exist in regulating fuel quality standards. In China, the Ministry of Ecology and Environment (MEE) is the leading governmental department in formulating and implementing vehicle emission standards, but it does not have the specific right to design the parameters of oil product quality, which directly affects vehicle emissions. The oil quality standard is created and managed by the National Petroleum Products and Lubricants Standardization Committee (also called TC280), and 43 of 67 members are directly related to the petrochemical industry while only 6 of 67 members are from the MEE. Obviously, the MEE cannot play a leading role if there are no “one ticket veto” rights. The representatives of petrochemical businesses dominate the technical committee. However, in many other countries, the establishment of national oil standards is usually hosted and dominated by relevant governmental departments. Fundamentally, the principle of environmental protection should be the highest priority when setting oil product quality standards (Li, 2015), while this priority has not yet been fully reflected in China. Comparatively, in the US, the EPA and the California Air Resources Board (CARB) have the authority to regulate vehicle emissions and fuel quality. The EPA and CARB can continue to renew vehicle emission standards in accordance with increasingly stringent fuel quality requirements. China’s MEE has limited influence on formulating oil product quality standards; therefore, it is much harder to upgrade fuel standards to tackle air pollution in China.

The sulfur (S) content is one of the most critical parameters in the fuel standard. The high content of sulfur can not only increase the emissions of toxic SOx but also make the three-way catalyst poisoned, resulting in growing emissions of CO, NOx, and VOCs, which are the important ingredients of smog in China (Geddes & Murphy, 2012). The sulfur content in oil has been a major target in updating fuel quality. China’s fuel standards have been gradually tightened since the late 1990s following changes in European standards. Compared with China IV gasoline (50 mg/kg sulfur), China III gasoline (150 mg/kg sulfur) emits much higher NOx (36%), CO (25%), and HC (13%) (Fung et al., 2011). It is important to note that the national sulfur limit has decreased from 1,000 mg/kg to 150 mg/kg for gasoline and declined from 10,000 mg/kg to ∼350 mg/kg for diesel (Tables 1 and 2). This implies that China has made considerable effort and progress in reducing sulfur content. However, there are large regional differences in fuel supply standards, which are generally encompassed by three: China III fuel standard (gasoline 150 mg/kg, diesel 350 mg/kg), China IV fuel standard (gasoline/diesel 50 mg/kg), and China V fuel standard (gasoline/diesel 10 mg/kg). Usually, in less developed regions, lower fuel supply standards are used. As the capital, Beijing implemented the first upgrades and reached China V gasoline fuel standard in May 2012. By 2014, half of the 135 cities in China had implemented China III emission standard (Zhang, 2015). For the diesel standard, although China has released the China IV emission standard in 2013, ordinary diesel is still used in most cities, and only a few cities have gradually begun to supply China III diesel (Wang & Hao, 2013).

**Table 1:** The implementation schedule of gasoline fuel standard (adapted from Zhang, 2015).

Sulfur content of gasoline fuels (mg/kg)												
	2001	2002	2003	2004	2005	2006	2007	2008	2009	2010	2011	2012
China	1,000		800		500					150 (China III)		
Beijing	800				500		150	50 (China IV)				10 (China V)
Europe	150				50				10			
Japan	100				50			10				
U.S.	150					30					15	

**Table 2:** The implementation schedule of automotive diesel fuels standard (adapted from Zhang, 2015).

Sulfur content of automotive diesel fuels (mg/kg)												
	2001	2002	2003	2004	2005	2006	2007	2008	2009	2010	2011	2012
China	2,000/5,000/10,000	2,000									350	
Beijing	2,000/5,000/10,000	2,000		500	350 (China III)			50 (China IV)				
Europe	350				50				10			

### Fuel standards and vehicle emission standards are not upgraded simultaneously

Optimal vehicle emission performance would be achieved if fuel and vehicle emission standards were implemented simultaneously. Unfortunately, the fuel standards have significantly lagged behind vehicle emission standards in many cities over the past decades in China. For example, the implementation of China III gasoline standards in January 2010 was two and a half years after the implementation of the China III light-duty vehicle standard. Furthermore, the implementation of China III diesel standards nationwide occurred in July 2011, four and a half years after the implementation of China III heavy-duty diesel vehicle standards. Using China III gasoline in China IV light-duty gasoline vehicles would not cause long-term damage to engines, but the emissions of NOx, CO, and HC can increase by 36%, 25%, and 13%, respectively, compared with the combustion of China IV gasoline (Fung et al., 2011). Some cities have supplied China IV gasoline, whereas there are still a large number of vehicles using the China IV emission standard. According to China’s annual report on motor vehicle pollution control, vehicles reaching or exceeding the China IV emission standard only accounted for 16% in 2013 (Ministry of Environmental Protection of the People’s Republic of China, 2013). Obviously, the lag in improving fuel quality and delayed implementation of more environmentally-friendly and stringent fuel standards have become a significant barrier to tackle vehicle emission-induced air pollution. Hence, oil product and vehicle emission standards should be upgraded simultaneously to achieve greater environmental benefits.

### Slow development of new energy vehicles

China’s strategy of new energy vehicles focuses on the development of pure electric, plug-in hybrid, and fuel cell vehicles. Regarding electric vehicles, China has achieved some success along with facing some challenges. Around 25 pilot cities have operated approximately 390,000 new energy vehicles, more than 80% of which are used for public services, primarily buses and taxis (Li et al., 2016). However, these public new energy vehicles, which are mainly in the form of battery electric vehicles (BEVs), are funded by government and usually operated with subsides. BEVs are considered to be the most promising because of their relatively simple and environment-friendly technology. However, the construction of charging stations and the sale of the new energy cars cannot maintain equal paces. Constructing and operating large-scale and commercial charging stations requires both tremendous investment and an effective operating model, so long-term and robust support from the government is required to ensure the competitiveness of BEVs (Safari, 2018). Furthermore, recharging BEVs takes a much longer time (usually several hours) than filling the tank of an ordinary fuel car (a few minutes). Additionally, the disadvantages of short battery life and high replacement price may limit the development of BEVs (Pettifor et al., 2017). Therefore, BEVs are still regarded as a transitional product, and the petrol-electric hybrid vehicle is more popular currently.

## Progress

The stalemate between the development of motor vehicles and the prevention/control of atmospheric pollution has gradually become a notable topic of discussion, particularly its severe effects on sustainable development and public health (Yang, 2014). After the US embassy released the PM_2.5_ data in Beijing in 2011 (Zhu, 2018), the Chinese Government has made increasing efforts for exploring some long-term and effective measures to mitigate air pollution, particularly air pollution from vehicles in cities. China’s State Council also ordered the local government to release the PM_2.5_ data to the public. Meanwhile, the revised Ambient Air Quality Standard added the average PM_2.5_ concentration limit and improved the requirement of PM_2.5_ management. Afterward, vehicle emission-induced air pollution control and supervision have become an important task to improve air quality. In 2012, the air quality improvement objectives concerning PM_2.5_ appeared on the central government’s report for the first time, and ecological protection was clarified as a human right. Furthermore, the MEE issued a guideline for central and local environmental protection departments to strengthen the prevention of motor vehicle pollution and boost the monitoring network of PM_2.5_. The progress of vehicle emission-induced air pollution prevention and control in China is categorized in the following aspects.

### The enhanced regulation and management of atmospheric pollution

The first milestone is the upgrade of atmospheric pollution related law. The Standing Committee of the National People’s Congress has revised the old version (1987) of the “Prevention and Control Law of Atmospheric Pollution” twice. These revisions directly led to more stringent vehicular emission standards as well as switching to non-leaded gasoline throughout the country, which largely decreased the pollutant emissions from vehicles (Shao et al., 2006). The second milestone is the General Bureau of State Environmental Protection Agency which was upgraded to the Ministry of Environmental Protection in March 2008 and then the Ministry of Ecology and Environment in March 2018 with unprecedentedly enhanced administrative power. Meanwhile, the revisions of atmospheric pollution related law grant MEE the right to regulate fuel parameters to control vehicle emissions (including sulfur levels). In other words, the MEE has been given more authority in formulating and implementing vehicle emission standards, even though it has not yet had the right to set fuel product standards. Hopefully, the MEE will be granted the right to establish fuel product standards in the near future.

### The release of national plan on energy saving and emission reduction

China’s State Council released “the 2014–2015 Low Carbon Development Plan for Energy Saving and Emission Reduction” in 2014, aiming to address the current situation of motor vehicle related air pollution. The Plan ordered that six million “yellow label” vehicles and old cars should be phased out by the end of 2014 and assigned this task to provincial governments. It laid a foundation for the promotion and implementation of the more stringent vehicle emission standards. The Plan demanded strengthening the environmental protection regulation on motor vehicles, especially on new vehicles whose environmental performance level should meet the latest requirement. The Plan also encourages the rapid and sustainable development of public transport and new energy vehicles. Overall, the Plan has provided a clear direction for the mitigation of motor vehicle emissions and improving air quality.

### Encouragement of “green travel”

“Green travel” includes the improvement of the public transport system and enhancement of the bus/metro priority development. With the increasing pressures from air pollution, the government has made it much clearer in recent years to prioritize the development of public transport in the urban traffic system (Zhang et al., 2017). Meanwhile, “Internet-based transportation” has led to the development of intelligent transportation, supporting the development of transportation infrastructure and improving convenient travel. For example, the development of intelligent terminals on mobile phones apps that focus on the user’s personal needs and experience, makes travel more convenient and intelligent. More importantly, the combination of electronic payment systems and intelligent transportation networks greatly improves the quality of travel services and users’ experiences, which is conducive to incentivizing the public to utilize public transportation and reduce the use of private vehicles.

### Impose restrictions on vehicle licensing

To control air pollution from vehicle emissions, transportation departments in some cities implemented the “one car and one license number” policy. By the end of 2014, seven cities including Shanghai, Beijing, Guiyang, Guangzhou, Tianjin, Hangzhou, and Shenzhen have implemented this policy to control the number of vehicles by limiting auto licensing. Beijing and Guiyang practiced the policy of license-plate lottery, Shanghai implemented the policy of vehicle license auctions, and other cities combined the policies of license-plate lottery and vehicle license auctions such as Tianjin, Guangzhou, Hangzhou, and Shenzhen (Huang, 2016). After several years of this special administrative measure, it becomes more difficult to acquire an auto license in these cities. This resulted in a gradually decreasing number of ordinary cars and increasing proportion of new energy vehicles on road.

### Phase out of “yellow label” vehicles

The elimination of “yellow label” cars and other old vehicles has been accelerated in recent years. Gasoline-fueled cars that do not reach China I emission standards or diesel-fueled vehicles that do not reach China III standard are classed as heavy-polluting vehicles, also known as “yellow label” cars. Around 13.494 million “yellow label” cars accounted for 10.7% of the national cars in 2013. However, their emissions of NOx, PM, CO, and HC accounted for 52.4%, 78.8%, 49.0% and 52.9% of the total emission, respectively (Wang & Hao, 2013). Therefore, the elimination of “yellow label” cars is essential for realizing emission reduction targets. The three primary measures to eliminate “yellow label” cars nationally include (i) offers of subsidies by the government and encouragement of owners to scrap “yellow label” cars; (ii) government-set schedules and defined areas where “yellow label” cars are conditionally permissible to run; and (iii) requests to retrofit emission control devices and acquire a “green label” after reaching emission standards as the responsibility of car owners. The strategy of eliminating “yellow label” and old cars has also been written in a government report, which pushes traffic administrative departments to phase out substandard cars more efficiently and effectively. An estimated total of 15 million yellow-label vehicles were scrapped between 2013 and 2015 (Liu et al., 2017a).

### Further promote new energy vehicles

In recent years, both the central and local governments have issued several policies and regulations concerning the sustainable development of new energy vehicles. The sale of new energy vehicles is not promising in recent years due to limitations including short battery life, inconvenience of energy supply, and high replacement costs, but governments are attempting to lower entry barriers to encourage more enterprises’ participation in market competition. Given such open policies and suitable market guidance, new energy automobile manufacturing companies and charging facilities manufacturing enterprises are endeavoring to break through the core technology. Meanwhile, enterprises with novel core technologies are encouraged to share technology and reduce their qualification admittance to solve the potential problem of monopolies (Yue et al., 2015). Unsurprisingly, new energy vehicles have been increasingly purchased by governments for public service. Furthermore, to propel the development of the new energy automotive industry and maintain the continuity of the policy, China Ministry of Finance announced that the subsidy policy of new energy automobile promotion will continue in 2016–2020 (Ministry of Finance of the People’s Republic of China, 2016). Consumers who purchase any battery electric vehicles (BEVs), fuel cell vehicles (FCVs), or plug-in hybrid electric vehicles (PHEVs) will be subsidized.

### Timely upgrade of fuel quality standards and emission standards

To date, China has upgraded fuel quality and emission standards to a highly advanced level that exceeds that of many other developing countries. Since the implementation of motor vehicle emission standards during the first phase (China I standard) in 2000, China has upgraded from China I to China V emission standards in 18 years. The full upgradation of the China V fuel standard was divided into two steps. First, in more advanced areas such as Beijing-Tianjin-Hebei, Yangtze River Delta, and Pearl River Delta, fuel standards were upgraded to China V by the end of 2015. Second, in the rest of China, the goal was meant to be achieved by the end of 2017 (Lin, 2014). According to the national standard committee, vehicle pollutant discharge would be significantly reduced after the implementation of China V in both fuel and emission standards. As expected, the consumption of China V gasoline will decrease roughly 300,000 tons of NOx emissions per year, while new cars can collectively reduce 90,000 tons of NOx emissions in five years (Zhang, 2014). China V emission standard is equivalent to Europe 5 emission standards. Vehicle emission assessment mileage is doubled in China V standard, and the emission control of the on-board diagnosis system is improved. Moreover, inspection requirements for key emission control components, such as a catalytic converter and canister, are enhanced (Ministry of Environmental Protection of the People’s Republic of China, 2013).

Currently, the MEE has planned to implement the emissions standard at stage 6 (China VI standard), which will not only follow European standards as before but also consider the experience of the US and California, in particular. Specifically, China VI emission standard contains more stringent emission limits than those in the Europe 6 regulations and adds a real-world emission testing protocol and 48 h evaporation testing procedure, including diurnal and hot soak emissions (Wu et al., 2017). China VI emission standard can mitigate total emissions of HC, CO, NO_X_, and PM_2.5_ by approximately 39%, 57%, 59%, and 79%, respectively, by 2030 (Wu et al., 2017).

### Toward participatory and transparent governance

Since 2010, the MEE has released vehicle emission data every year. The government publishes the monthly top and bottom ten cities ranked by air quality and updates the hourly air quality monitoring data of more than 70 cities online (Ministry of Environmental Protection of the People’s Republic of China, 2010). The government even held a press conference through a micro-blog to discuss the problem of air pollution. The unexpected rapid transparency of air quality data was welcomed by the Chinese people and international organizations (Xinhua News Agency, 2013).

The MEE urged Beijing, Tianjin, Shanghai, Hebei, Liaoning, Sichuan, Fujian, Guangdong, Hunan, Hubei, and 13 other provinces and cities to conduct pilot work for compiling the emission inventory in 2015. The emission inventory includes nine pollutants SO_2_, NOx, CO, VOC, NH_3_, PM_10_, PM_2.5_, BC, and OC (Ministry of Environmental Protection of the People’s Republic of China, 2016). Compiling such a list is conducive for understanding pollutant concentration and emission sources in the atmospheric environment, providing an important basis for the formulation of air pollutant emission reduction targets, and preparing an emergency plan for heavily polluted areas.

## Prospects

Air pollution associated with the booming expansion of vehicles market and consumption has already received increasing attention from the public. There is a range of rising pressures to control vehicle emissions in China due to many aspects such as the huge number of on-road vehicles, slow development of new energy vehicles, slow improvement of fuel, and overall shortage of efficient highway systems. Thus, a variety of measures have been considered and implemented continually, which have temporarily yielded considerable achievements. To attain the long-term benefits of vehicle industry development and sound air quality, an integrated mechanism of social participation, technical revolution, and regulatory innovation in vehicles, fuel, and roads is suggested as follows.

### Vehicle

China must further expedite the elimination of “yellow label” vehicles. Local governments should accomplish the phasing out of “yellow label” vehicles as soon as possible. To achieve this goal, collaboration between the government, enterprises, and consumers is urgently required. The government must consider the impact of this action rather than simply focus on immediate conditions (Yang, Flower & Thompson, 2013). For example, in the early 1970s, the serious problem of illegally dumped scrapped vehicles troubled in Japan. In order to establish and regulate the vehicle recycling industry, the Japanese Government passed “the Automobile Recycling Law” which was the first law to address vehicle recycling. It outlined the process of scrapping vehicles throughout the whole process of design, manufacture, and production (Lu, 2007). Inevitably, if consumers are able to obtain a larger refund from the second-hand vehicle market or non-certified vehicle dismantling company, they would not want to sell scrap vehicles to a qualified vehicle dismantling company. Therefore, local governments can consider granting rewards and subsidies to those who give up their cars in advance and encouraging vehicle companies and financial institutions to give discounts or provide low-interest loans to those who discard “yellow label” vehicles and buy a new car.

On the other hand, in order to enhance environmental inspections of in-use vehicles, governments should not only strengthen the regular inspection of vehicles at larger scale, but also encourage the public to public transportation more frequently. Emissions could be recorded as an important part of the annual check. Vehicles that are substandard on emissions should not be issued with a qualified environmental protection logo. The environmental protection department should heavily punish owners with unmet emission standard vehicles. Moreover, it is quite important to further promote the development of new energy vehicles with significant policy support.

For the long-term development of new energy vehicles, there is an increasing necessity for the enhanced cooperation and integration of different stakeholders. The government could better guide and integrate funds and resources from society to develop core technologies for new energy vehicles. In other words, the government should cultivate an innovative and competitive environment for the new energy vehicle industry through flexible policies and effective cooperation between enterprises and other stakeholders. In addition, new energy vehicles should be addressed differently in car quota indicators and license quota auctions and be subsidized with lower parking fees. Enterprises should bear more social responsibility and be encouraged to reform, especially for traditional nation-owned car companies. Moreover, they must understand consumer’s needs (convenience, security, and sound after-sales service) when promoting the new energy cars. The development of low carbon or non-fossil electricity is also crucial (Yang, Clarke & Thompson, 2016; Yang & Thompson, 2014).

### Fuel

China should further improve vehicle fuel standards in line with the national refinement of industry conditions. It is advised that the olefins, aromatics, and octane labels in gasoline should be established within a reasonable range to avoid excessive waste of industrial investment due to over-upgrading. Biofuel and other renewable energy could be used as alternative to vehicle fuel for mitigating air pollution. Renewable clean fuels—for example, ethanol, bio-diesel, and other biofuels—should be promoted with long-term stability. Particularly, non-plantation resource-based biofuel should receive more attention so that plantation resource-based biofuel may be developed with more caution due to the possible competition of land use with other sectors (Hao et al., 2018). Compared with traditional fossil fuel, technology innovation is still the key to improving the market competitiveness of renewable energies. It is also important to establish regulations and laws intended to promote the renewable energy industry, which can effectively encourage China’s various stakeholders to be involved in and support the development of renewable energies.

For the heavily polluted areas, the supervision and inspection of fuel quality throughout production and distribution should be further strengthened. In particular, Beijing-Tianjin-Hebei, Yangtze River Delta, Pearl River Delta, and other heavily polluted regions should implement more stringent measures to address the import and sale of substandard oil. Furthermore, authorities should enhance *in-situ* checks on fuel production enterprises and gas stations, ensuring the punishment of illegal acts, and establishing blacklists of illegal enterprises to ensure that the punishment system is transparent (Yang et al., 2015).

### Road

Road network planning should be further optimized. In the planning of comprehensive transport, the connecting system of non-motorized lanes and public transportation should be designed to ensure sustainable development of urban traffic (Yuan, 2010). The experiences of many large cities like Hong Kong could be good examples. Hong Kong has begun to mitigate air pollution much earlier than cities in mainland China. Through improving urban planning, adding convenient and high-quality public transit, and charging high parking fees for certain areas, Hong Kong has greatly reduced the air pollution (Zhao & Melaina, 2006). Cities such as Beijing, Shanghai, and Guangzhou can certainly learn from Hong Kong’s experience and further improve the air quality. Currently, the city transit trip ratio in mainland China is around 30–40%, which is still fairly low compared with Hong Kong, Tokyo, Paris, and other foreign cities (which are more than 70–80%) (Zhang & Wu, 2015). To achieve the sustainable development of urban traffic and the goal of mitigating air pollution, it is crucial to reduce reliance on private cars, minimize traffic congestion, and further improve the efficiency of short travel by enhancing “slow traffic” and public traffic capabilities, in particular, the construction of higher-class highways. Thus, the development of urban public transport systems with low carbon, high efficiency, and large capacity can adequately control the traffic volume, which will in turn mitigate air pollution.

## Conclusion

With the soaring development of China’s vehicle market, the vehicle-related air pollution has become increasingly serious. Frequent air pollution in many Chinese cities has mainly attributed to the rocketing number of vehicles on road. With an attempt to create a green, harmonious, and sustainable transportation system, an integrated mechanism of social participation, technical revolution, and regulatory innovation in vehicles, fuel, and roads has been highlighted. It recommends more stringent emission standards in line with updated fuel standards, advanced new energy vehicles, and more reasonable and mature infrastructure-traffic systems. In recent years, the Chinese Government has made great efforts through a series of stringent and preventive measures, better mitigate vehicle emissions, and alleviate air pollution. Various endeavors from different stakeholders in the context of the rapidly increasing numbers of vehicles have yielded positive effects. Even though new challenges are unavoidable in the implementation of stricter policies, China must establish measures that are more appropriate to address vehicle associated air pollution. Notably, China will continue to develop more stringent fuel and emission standards toward the effective control of vehicle induced air pollution and achieve long lasting social and environmental benefits from integrated mechanism and policies.

## Additional Information and Declarations

### Data Availability

The following information was supplied regarding data availability: This is a review paper and does not include any raw data or code.

## References

[ref-1] Attfield MD, Schleiff PL, Lubin JH, Blair A, Stewart PA, Vermeulen R, Coble JB, Silverman DT (2012). The diesel exhaust in miners study: a cohort mortality study with emphasis on lung cancer. Journal of the National Cancer Institute.

[ref-2] Bai L, Su X, Zhao D, Zhang Y, Cheng Q, Zhang H, Wang S, Xie M, Su H (2018). Exposure to traffic-related air pollution and acute bronchitis in children: season and age as modifiers. Journal of Epidemiology and Community Health.

[ref-3] Bell JNB, Honour SL, Power SA (2011). Effects of vehicle exhaust emissions on urban wild plant species. Environmental Pollution.

[ref-4] Che WW, Zheng JY, Wang SS, Zhong LJ, Lau AI (2011). Assessment of motor vehicle emission control policies using Model-3/CMAQ model for the Pearl River Delta region, China. Atmospheric Environment.

[ref-5] Cherrie JW, Apsley A, Cowie H, Steinle S, Mueller W, Lin C, Horwell CJ, Sleeuwenhoek A, Loh M (2018). Effectiveness of face masks used to protect Beijing residents against particulate air pollution. Occupational and Environmental Medicine.

[ref-6] Chinese Automotive Technology & Research Centre (2015). Annals report on the development of China automotive industry 1994–2014.

[ref-7] Chinese Automotive Technology & Research Centre (2017). Annals report on the development of China automotive industry 2015–2016.

[ref-8] Feng YY, Chen SQ, Zhang LX (2013). System dynamics modeling for urban energy consumption and CO_2_ emissions: a case study of Beijing, China. Ecological Modelling.

[ref-9] Fung F, He H, Sharpe B, Kamakaté F, Blumberg K (2011). Overview of China’s vehicle emission control program past success and future prospects.

[ref-10] Geddes JA, Murphy JG (2012). The science of smog: a chemical understanding of ground level ozone and fine particulate matter. Metropolitan sustainability.

[ref-11] Gong MM, Yin SS, Gu XK, Xu YQ, Jiang N, Zhang RQ (2017). Refined 2013-based vehicle emission inventory and its spatial and temporal characteristics in Zhengzhou, China. Science of the Total Environment.

[ref-12] Guo XR, Fu LW, Ji MS, Lang JL, Chen DS, Cheng SY (2016). Scenario analysis to vehicular emission reduction in Beijing-Tianjin-Hebei (BTH) region, China. Environmental Pollution.

[ref-13] Hao J, He D, Wu Y, Fu L, He K (2000). A study of the emission and concentration distribution of vehicular pollutants in the urban area of Beijing. Atmospheric Environment.

[ref-14] Hao H, Liu Z, Zhao F, Ren J, Chang S, Rong K, Du J (2018). Biofuel for vehicle use in China: Current status, future potential and policy implications. Renewable and Sustainable Energy Reviews.

[ref-15] He K, Huo H, Zhang Q (2002). Urban air pollution in China: current status, characteristics, and progress. Annual Review of Energy and Environment.

[ref-16] Huang Y (2016). Research on the legality of “limited licensing policy” doctor.

[ref-17] Huang WQ, Fan HB, Qiu YF, Cheng ZY, Xu PR, Qian Y (2016a). Causation mechanism analysis for haze pollution related to vehicle emission in Guangzhou, China by employing the fault tree approach. Chemosphere.

[ref-18] Huang X, Wang Y, Xing Z, Du K (2016b). Emission factors of air pollutants from CNG-gasoline bi-fuel vehicles: Part II. CO, HC and NOx. Science of the Total Environment.

[ref-19] Li KS (2015). Who should lead the development of oil standards?. Environmental Economy.

[ref-20] Li JB, Huang XJ, Kwan MP, Yang H, Chuai XW (2018). The effect of urbanization on carbon dioxide emissions efficiency in the Yangtze River Delta, China. Journal of Cleaner Production.

[ref-21] Li L, Xie S (2014). Historical variations of biogenic volatile organic compound emission inventories in China, 1981–2003. Atmospheric Environment.

[ref-22] Li Y, Zhan CJ, De Jong M, Lukszo Z (2016). Business innovation and government regulation for the promotion of electric vehicle use: lessons from Shenzhen, China. Journal of Cleaner Production.

[ref-23] Lin BQ (2014). Oil upgrade schedule: 2017 national standard V standard. Vehicle Maintenance.

[ref-24] Liu F, Beirle S, Zhang Q, Zheng B, Tong D, He K (2017a). NOx emission trends over Chinese cities estimated from OMI observations during 2005 to 2015. Atmospheric Chemistry and Physics.

[ref-25] Liu YH, Liao WY, Lin XF, Li L, Zeng XL (2017c). Assessment of co-benefits of vehicle emission reduction measures for 2015–2020 in the Pearl River Delta region, China. Environmental Pollution.

[ref-26] Liu L, Wu T, Li S, De Jong M, Sun Y (2017b). The drivers of local environmental policy in China: an analysis of Shenzhen’s environmental performance management system, 2007–2015. Journal of Cleaner Production.

[ref-27] Lu W (2007). Implementation of the scientific outlook on development, vigorously developing circular economy. China’s National Strength.

[ref-28] Lv Y, Huang G, Li Y, Yang Z, Sun W (2012). Development of a sequential decision-making model for controlling multiple air pollutants under stochastic uncertainty. Water, Air, & Soil Pollution.

[ref-29] Meng H, Huang X, Yang H, Chen Z, Yang J, Zhou Y, Li J (2018). The influence of local officials’ promotion incentives on carbon emission in Yangtze River Delta, China. Journal of Cleaner Production.

[ref-30] Ministry of Environmental Protection of the People’s Republic of China (2010). Calendar in Nov. 2010. http://www.mee.gov.cn/gkml/sthjbgw/qt/201011/t20101104_197140.htm?keywords=.

[ref-31] Ministry of Environmental Protection of the People’s Republic of China (2013). Limits and measurement methods for emissions from lightweight vehicles: China’s fifth stage (GB 18352.5—2013).

[ref-32] Ministry of Environmental Protection of the People’s Republic of China (2016). China vehicle emission control annual report 2010–2015.

[ref-33] Ministry of Environmental Protection of the People’s Republic of China (2017). China vehicle environmental management annual report 2016.

[ref-34] Ministry of Finance of the People’s Republic of China (2016). Notice on adjusting the policy of promoting and applying financial subsidies for new energy vehicles. Governmental Report, Document number: 2016–958.

[ref-35] Pettifor H, Wilson C, McCollum D, Edelenbosch OY (2017). Modelling social influence and cultural variation in global low-carbon vehicle transitions. Global Environmental Change-Human and Policy Dimensions.

[ref-36] Safari M (2018). Battery electric vehicles: looking behind to move forward. Energy Policy.

[ref-37] Samaras Z, Sorensen SC, Fenger J, Hertel O, Palmgren F (1998). Mobile sources. Urban air pollution—European aspects.

[ref-38] Shao M, Tang XY, Zhang YH, Li WJ (2006). City clusters in China: air and surface water pollution. Frontiers in Ecology and the Environment.

[ref-39] Song S (2014). China’s clean air challenge: the health impacts of transport emissions. http://www.wri.org.cn/en/node/41165.

[ref-40] Song Z, Li R, Qiu R, Liu S, Tan C, Li Q, Ge W, Han X, Tang X, Shi W, Song L, Yu W, Yang H, Ma M (2018). Global land surface temperature influenced by vegetation cover and PM2.5 from 2001 to 2016. Remote Sensing.

[ref-41] Sun Y (2017). The real monthly car sales in China. Operator: Automotive Business Review.

[ref-42] Van Wijnen J, Van der Zee S (1998). Traffic-related air pollutants: exposure of road users and populations living near busy roads. Reviews on environmental health.

[ref-43] Vedal S, Han B, Xu J, Szpiro A, Bai Z (2017). Design of an air pollution monitoring campaign in beijing for application to cohort health studies. International Journal of Environmental Research and Public Health.

[ref-44] Wang GG, Hao TQ (2013). Analysis of China’s gasoline product quality upgrade status. Guangdong Chemical Industry.

[ref-45] Wang SX, Zhao M, Xing J, Wu Y, Zhou Y, Lei Y, He KB, Fu LX, Hao JM (2010). Quantifying the air pollutants emission reduction during the 2008 olympic games in Beijing. Environmental Science & Technology.

[ref-46] Wang T, Xie S (2009). Assessment of traffic-related air pollution in the urban streets before and during the 2008 Beijing Olympic Games traffic control period. Atmospheric Environment.

[ref-47] Wong H (2013). 2013: the year deadly, suffocating smog consumed China. https://www.citylab.com/equity/2013/12/2013-year-deadly-suffocating-smog-consumed-china/7925/.

[ref-48] Wu Z, Shan H, Wang Y (2014). Using underground space construction to improve the city slow traffic system. Huazhong Architecture.

[ref-49] Wu XM, Wu Y, Zhang SJ, Liu H, Fu LX, Hao JM (2016). Assessment of vehicle emission programs in China during 1998–2013: achievement, challenges and implications. Environmental Pollution.

[ref-50] Wu Y, Zhang SJ, Hao JM, Liu H, Wu XM, Hu JN, Walsh MP, Wallington TJ, Zhang KM, Stevanovic S (2017). On-road vehicle emissions and their control in China: a review and outlook. Science of the Total Environment.

[ref-51] Xinhua News Agency (2013). Foreign media: China face haze frankly and transparently. http://china.cankaoxiaoxi.com/2013/0116/151585.shtml.

[ref-52] Xu W (2000). Urban traffic planning theory.

[ref-53] Xu L, Zhou J, Guo Y, Wu T, Chen T, Zhong Q, Yuan D, Chen P, Ou C (2017). Spatiotemporal pattern of air quality index and its associated factors in 31 Chinese provincial capital cities. Air Quality, Atmosphere & Health.

[ref-54] Yang H (2014). China must continue the momentum of green law. Nature.

[ref-55] Yang H, Clarke JL, Thompson JR (2016). Nuclear energy: improve collaboration. Science.

[ref-56] Yang H, Flower RJ, Thompson JR (2013). China’s new leaders offer green hope. Nature.

[ref-57] Yang H, Flower R, Thompson J (2018). Identify and punish ozone depleters. Nature.

[ref-58] Yang H, Huang X, Thompson JR, Bright RM, Astrup R (2016). The crushing weight of urban waste. Science.

[ref-59] Yang H, Huang X, Thompson JR, Flower RJ (2014). Soil pollution: urban brownfield. Science.

[ref-60] Yang H, Huang X, Thompson JR, Flower RJ (2015). Enforcement key to China’s environment. Science.

[ref-61] Yang H, Ma M, Thompson JR, Flower RJ (2018). Waste management, informal recycling, environmental pollution and public health. Journal of Epidemiology & Community Health.

[ref-62] Yang H, Thompson JR (2014). Shale gas is a fraught solution to emissions. Nature.

[ref-63] Yin SS, Zheng JY, Lu Q, Yuan ZB, Huang ZJ, Zhong LJ, Lin H (2015). A refined 2010-based VOC emission inventory and its improvement on modeling regional ozone in the Pearl River Delta Region, China. Science of the Total Environment.

[ref-64] Yuan J (2010). Research on planning method of non-motorized system in city roads. Marster Dissertation.

[ref-65] Yue X, Wu Y, Hao J, Pang Y, Ma Y, Li Y, Li B, Bao X (2015). Fuel quality management versus vehicle emission control in China, status quo and future perspectives. Energy Policy.

[ref-66] Zhang XM (2014). The rough road of state IV. Special Purpose Vehicle.

[ref-67] Zhang B (2015). State Council: speed up oil upgrades and promote air pollution control. China Auto Parts Market.

[ref-68] Zhang QT, Gong XQ (2004). Ecological crisis and countermeasures of urbanization in China. Journal of Market Economy.

[ref-69] Zhang L, Long R, Chen H, Geng J (2018). A review of China’s road traffic carbon emissions. Journal of Cleaner Production.

[ref-70] Zhang YB, Wu XQ (2015). China’s oil standards are low, the sulfur content is equivalent to Europe and the United States a decade ago. http://data.163.com/15/0130/00/AH5S6ILD00014MTN.html.

[ref-71] Zhang SJ, Wu Y, Zhao B, Wu XM, Shu JW, Hao JM (2017). City-specific vehicle emission control strategies to achieve stringent emission reduction targets in China’s Yangtze River Delta region. Journal of Environmental Sciences.

[ref-72] Zhao J (2014). Corporate social responsibility in contemporary China.

[ref-73] Zhao J, Melaina MW (2006). Transition to hydrogen-based transportation in China: lessons learned from alternative fuel vehicle programs in the United States and China. Energy Policy.

[ref-74] Zheng JY, Zhang LJ, Che WW, Zheng ZY, Yin SS (2009). A highly resolved temporal and spatial air pollutant emission inventory for the Pearl River Delta region, China and its uncertainty assessment. Atmospheric Environment.

[ref-75] Zhu W (2018). Role of PM2. 5 (particulate matter) event in the formation of a green public sphere in China. Applied Environmental Education & Communication.

[ref-76] Zong R, Yang X, Wen L, Xu C, Zhu Y, Chen T, Yao L, Wang L, Zhang J, Yang L (2018). Strong ozone production at a rural site in the North China Plain: mixed effects of urban plumes and biogenic emissions. Journal of Environmental Sciences.

